# Factors Governing the Erythropoietic Response to Intravenous Iron Infusion in Patients with Chronic Kidney Disease: A Retrospective Cohort Study

**DOI:** 10.3390/biomedicines11092417

**Published:** 2023-08-29

**Authors:** Chukwuma A. Chukwu, Helen Gilbody, Olivia Wickens, Craig Carroll, Sunil Bhandari, Philip A. Kalra

**Affiliations:** 1 Department of Nephrology Salford Royal Hospital, Northern Care Alliance NHS Foundation Trust, Salford M6 8HD, UK; olivia.wickens@panthera-bio.com (O.W.); craig.carroll@nca.nhs.uk (C.C.); philip.kalra@nca.nhs.uk (P.A.K.); 2 Faculty of Biology, Medicine and Health, University of Manchester, Manchester M13 9PL, UK; 3 College of Medical and Dental Sciences, University of Birmingham, Birmingham B15 2TT, UK; hmg944@student.bham.ac.uk; 4 Academic Renal Research Department, Hull University Teaching Hospitals NHS Trust and Hull York Medical School, Kingston upon Hull, Hull HU3 2JZ, UK; sunil.bhandari@nhs.net

**Keywords:** iron deficiency anaemia, chronic kidney disease, functional iron deficiency, absolute iron deficiency, iron infusion

## Abstract

Background: Limited knowledge exists about factors affecting parenteral iron response. A study was conducted to determine the factors influencing the erythropoietic response to parenteral iron in iron-deficient anaemic patients whose kidney function ranged from normal through all stages of chronic kidney disease (CKD) severity. Methods: This retrospective cohort study included parenteral iron recipients who did not receive erythropoiesis-stimulating agents (ESA) between 2017 and 2019. The study cohort was derived from two groups of patients: those managed by the CKD team and patients being optimised for surgery in the pre-operative clinic. Patients were categorized based on their kidney function: Patients with normal kidney function [estimated glomerular filtration rate (eGFR) ≥ 60 mL/min/1.73 m^2^] were compared to those with CKD stages 3–5 (eGFR < 60 mL/min/1.73 m^2^). Patients were further stratified by the type of iron deficiency [absolute iron deficiency (AID) versus functional iron deficiency (FID)]. The key outcome was change in hemoglobin (∆Hb) between pre- and post-infusion haemoglobin (Hb) values. Parenteral iron response was assessed using propensity-score matching and multivariate linear regression. The impact of kidney impairment versus the nature of iron deficiency (AID vs. FID) in response was explored. Results: 732 subjects (mean age 66 ± 17 years, 56% females and 87% White) were evaluated. No significant differences were observed in the time to repeat Hb among CKD stages and FID/AID patients. The Hb rise was significantly lower with lower kidney function (non-CKD and CKD1–2; 13 g/L, CKD3–5; 7 g/L; *p* < 0.001). When groups with different degrees of renal impairment were propensity-score matched according to whether iron deficiency was due to AID or FID, the level of CKD was found not to be relevant to Hb responses [unmatched (∆Hb) 12.1 vs. 8.7 g/L; matched (∆Hb) 12.4 vs. 12.1 g/L in non-CKD and CKD1–2 versus CKD3–5, respectively]. However, a comparison of patients with AID and FID, while controlling for the degree of CKD, indicated that patients with FID exhibited a diminished Hb response regardless of their level of kidney impairment. Conclusion: The nature of iron deficiency rather than the severity of CKD has a stronger impact on Hb response to intravenous iron with an attenuated response seen in functional iron deficiency irrespective of the degree of renal impairment.

## 1. Introduction

Iron deficiency anaemia (IDA) can be the result of reduced iron intake, reduced intestinal absorption or chronic blood loss resulting in absolute iron deficiency (AID). In addition to this, a state of functional iron deficiency (FID) occurs when there is inadequate iron available for erythropoiesis in the presence of adequate iron stores [1]. This state is commonly associated with inflammation [2,3].

In chronic kidney disease (CKD), apart from being an independent risk factor for death, IDA is associated with reduced quality of life, cardiovascular disease (CVD), hospitalizations, and cognitive impairment [4,5,6]. Pre-operative IDA has been associated with higher peri- and postoperative morbidity and mortality [7,8].

Iron supplementation in IDA reduces the need for blood transfusion, length of hospital stays and the costs to healthcare providers [9]. In CKD, it reduces the requirement for erythropoiesis-stimulating agents (ESA) [10]. Over the years, intravenous infusions of iron have evolved from a poorly effective and relatively dangerous intervention to a safe cornerstone in the treatment of iron deficiency [11]. Modern iron formulations comprising composite nanoparticle carbohydrate ferric oxy-hydroxides have proven to be safe, so they can be infused over up to one hour at high doses, thereby allowing the correction of total iron deficit with single or repeated doses in 1–2 weeks depending on the formulation [11]. During the management of IDA, it is important to differentiate between AID and FID, as pathophysiology and response to therapy differ between these two conditions [12,13]. 

CKD is classified into five stages based on the estimated glomerular filtration rate (eGFR) and the presence of kidney damage. CKD Stages 1 (eGFR ≥ 90 mL/min/1.73 m^2^) and 2 (eGFR 60–89 mL/min/1.73 m^2^) represent preserved kidney function but require the presence of albuminuria or other evidence of kidney damage for CKD diagnosis. CKD Stages 3–5 encompass a range of impaired kidney function levels, graded by progressively reduced filtration capacity [CKD Stage 3a: eGFR 45–59; 3b: eGFR 30–44; Stage 4: severe reduction in kidney function, eGFR 15–29; and CKD Stage 5 (end-stage renal disease, ESRD) eGFR < 15 mL/min/1.73 m^2^ or the need for kidney replacement therapy (dialysis or kidney transplant)]. 

Current management guidelines recommend parenteral iron for anemic CKD5 hemodialysis patients and non-dialysis CKD3–5 patients with severe iron deficiency, inadequate response, or oral iron intolerance [14,15]. It is worth noting that although both AID and FID can be present in CKD, FID is a more predominant finding in CKD patients. 

In the perioperative setting, parenteral iron is recommended for patients that are intolerant of or non-adherent to oral iron or when the preoperative period is too short for oral iron to effectively correct the pre-operative anaemia and/or iron deficiency [9,16]. 

Presently, none of the accessible laboratory tests possess sufficient discriminatory power to differentiate patients will have a favorable hemoglobin (Hb) response to parenteral iron from those likely to have a sub-optimal response. [17].

The pre-operative anaesthetic department at our institution noticed that surgical patients with lower eGFR exhibited a less favorable response to parenteral iron compared to those with well-preserved eGFR (>60 mL/min/1.73 m^2^). Determining the validity of and explanation for this observation underpinned the rationale for the current study. This retrospective observational study was therefore conducted to evaluate the response to parenteral iron in patients with differing degrees of kidney function and to identify reasons for differential response.

## 2. Materials and Methods

### 2.1. Study Design, Setting and Participants

This was a retrospective cohort study of subjects who received parenteral iron in the form of ferric derisomaltose (Monofer^®^; Pharmacosmos A/S, Holbaek, Denmark) at a large hospital that included a tertiary nephrology centre in northwest England between 2017 and 2019. The study cohort was derived from two patient groups merged into a single cohort. A total of 81% of the iron infusions were delivered to patients in the CKD clinics, whereas the remaining 19% were in pre-operative patients who received parenteral iron for IDA. Subjects were excluded if they were receiving ESA or had no post-infusion Hb recorded within 90 days of infusion. 

Data were derived from electronic patient records and included patient demographics such as age, gender, ethnicity, and BMI. Data on patient comorbidities included history of diabetes and cardiovascular diseases (defined as a composite of heart failure, ischemic heart disease, stroke, and peripheral vascular disease). The underlying kidney disease was recorded for the CKD patients, and the nature of planned surgery was recorded for the surgical patients. The severity of CKD based on the stages of CKD was also recorded. Data on baseline and post infusion Hb parameters, iron indices and red-cell indices as well as the parenteral iron dose were also recorded. All data were fully anonymized.

### 2.2. Definition of Anaemia, FID and AID

Anaemia was defined as Hb < 130 g/L for men and <120 g/L for women according to World Health Organisation definitions [18].

Based on their iron parameters, patients were categorized into those with FID or AID. 

Two different definitions of FID and AID were used in this study. This was because the FID definition differs between CKD and non-CKD populations due to the influence of CKD and chronic inflammation on iron metabolism [19]. Since our cohort consisted of both CKD and non-CKD patients, we included both definitions in our analysis.

- According to the international consensus statement on the perioperative management of anaemia and iron deficiency, AID was defined as ferritin < 30 µg/L, whereas FID was defined as ferritin ≥ 30 µg/L and Tsat < 20%.
- In the CKD3–5 population, AID was defined as ferritin ≤ 100 µg/L and Tsat < 20%, whereas FID was defined as ferritin > 100 µg/L with Tsat < 20%.

### 2.3. Iron Infusion Protocol

The total dose of iron (TDI) infused for each infusion episode was also recorded. A simplified dosing regime, based upon Hb and weight and differing between the pre-operative and CKD clinics, was used as shown in Table 1. In patients with less severe anaemia, their TDI was administered in a single infusion. In contrast, some patients with more severe anaemia required a higher TDI, and, with the need for maximal dosing of 20 mg/kg, they received their infusion in two divided doses one week apart (Table 1). In the analysis, the cohort was also divided into tertiles based on the received iron dose per kg body weight. 

Iron infusion-associated reactions were categorized into mild, moderate, and severe reactions:

- Mild reactions: defined as localized infusion site reactions that did not result in discontinuation of infusions.
- Moderate reactions; defined as systemic symptoms leading to discontinuation of infusions but not to hospitalization.
- A severe reaction; defined as systemic symptoms requiring hospitalization for ≥ 24 h.

### 2.4. Ethical Permissions

The study procedure was compliant with the principles of the declaration of Helsinki and was approved by the local institutional review board—the Northern Care Alliance NHS Group (Ref: S21HIP50). As this was a retrospective observational study using routinely collected and fully anonymized data, the need for individual patient consent was not considered necessary.

### 2.5. Outcomes and Predictor Variables

The primary outcome was the change in Hb (∆Hb) post-parenteral iron, defined as the difference between the pre-infusion Hb and the post-infusion Hb. This was first compared across the various stages of CKD. Then we compared the response between subjects with FID and those who had AID. To address potential sources of bias, a propensity-score matching (PSM) method was used to balance out the effect of CKD severity and other baseline variables recognized for their impact on hemoglobin response. This enabled us to disentangle and evaluate the influence of the type of iron deficiency on the observed response isolating it from the influence of CKD on response and vice versa. A multivariate linear regression analysis was also conducted to determine the wider predictors of Hb response.

### 2.6. Statistical Analysis

Descriptive statistics of categorical data were summarized as frequencies and percentages. Continuous variables were summarized as the mean ± standard deviation (SD) or median and interquartile range (IQR). Inter-group comparisons were made using a one-way ANOVA or the Kruskal–Wallis rank test. The Chi-square or Fisher’s exact test was used to compare categorical variables. 

### 2.7. Propensity Scores Matched (PSM) Analysis 

Separate PSM analyses were undertaken to provide clearer insights into the effects of kidney function, FID and AID on the response to parenteral iron. In the initial set of PSM analyses, separate propensity scores (PS) were generated for matching subjects within the following groups:

- eGFR ≥ 60 mL/min/1.73 m^2^ (as the treated group) vs. eGFR < 60 mL/min/1.73 m^2^ (nominally termed the ‘control’ group)
- eGFR ≥ 60 mL/min/1.73 m^2^ (treated) vs. eGFR < 30 mL/min/1.73 m^2^ (control group)
- eGFR = 30–59 mL/min/1.73 m^2^ (treated) vs. eGFR < 30 mL/min/1.73 m^2^ (control group).

PS were calculated using the following variables: age, gender, diabetes, baseline Hb, baseline ferritin, baseline Tsat, presence or absence of FID and time from iron infusion to repeat Hb. In the second PSM analysis, separate PS models were used to assess the effect of the two types of IDA AID (treated group) vs. FID (control group). To ensure that subjects met the definitions of FID, those with both normal ferritin and Tsat (>20%) were excluded from the inferential analysis. The PS were estimated using the following variables: age, gender, diabetes, baseline Hb, baseline eGFR and time to repeat Hb. To assess the effect of kidney impairment on ∆Hb, the type of iron deficiency was included in the covariates used to generate PS. Conversely, to evaluate the impact of the nature of iron deficiency on ∆Hb, the degree of kidney impairment (baseline eGFR) was one of the covariates used to generate PS. This minimised the confounding effect of kidney impairment while evaluating the effect of FID on response and vice versa. 

PSM was conducted with radius matching (see Supplementary Information and Supplementary Table S1). 

### 2.8. Multivariate Regression Analysis

The wider predictors of response to parenteral iron were assessed using multivariate linear regression. Predictor covariates were chosen based on previous knowledge of their effect on anaemia, iron metabolism and erythropoiesis. They included age, gender, ethnicity, body mass index (BMI), diabetes, CVD, TDI tertile, baseline eGFR, baseline mean corpuscular volume (MCV) and the nature of iron deficiency (FID vs. AID). The time from infusion to Hb recheck was also included in the multivariate linear regression model. This addition aimed to mitigate potential bias stemming from variations in the duration before Hb recheck post-infusion. Furthermore, to avoid the potential confounding of Hb response by inadequate total parenteral iron dose, the total iron deficit for each subject was calculated using the Ganzoni formula [20]. We then proceeded to examine the ∆Hb among patients who received a parenteral iron dose that closely matched their calculated iron deficit (within a range of 200 mg from the calculated iron requirement). Statistical analysis was performed with Stata software version 14 licensed to the University of Manchester.

## 3. Results

The baseline characteristics of the subjects are presented in Table 2. A total of 1341 iron infusion episodes were evaluated in 952 subjects. Out of these, 507 infusions were excluded, 155 because Hb was not checked within 90 days of infusion, 323 infusions because the patient was receiving ESA, and for 29 cases the iron was never administered. Hence the final analysis included 834 iron infusions administered to 732 subjects (673 infusions in 602 CKD patients and 161 infusions in 130 pre-operative patients). The mean age of the subjects was 66 ± 17 years, with an age range of 18 to 98 years. Among the participants, 56% were female, 87% were of white ethnicity, 41% had diabetes, and 36% had cardiovascular disease. Notably, 81% (*n* = 673) of infusions originated from CKD iron clinics, while 19% (*n* = 161) were from pre-operative iron clinics.

The median total iron dose per kilogram of body weight was 18.0 mg/kg (interquartile range: 15–21 mg/kg). Most subjects (88%) received their complete iron dose as a single infusion, while 12% required two infusions spaced one week apart.

Regarding the distribution of subjects based on kidney function, the largest proportion (47%) fell into CKD stage 4 (*n* = 398), followed by those with well-preserved eGFR (eGFR > 60 mL/min/1.73 m^2^ either without CKD or CKD stages 1–2) accounting for 25% (*n* = 191). A small subset of patients (*n* = 6) was receiving renal replacement therapy. Baseline median Hb was 107.0 g/L (interquartile range: 99.0–116.0 g/L) with higher baseline Hb associated with higher kidney function. Conversely, baseline median ferritin was 92.0 µg/L (interquartile range: 33.0–211.5 µg/L), and higher ferritin was associated with worse CKD stage.

In accordance with the classical definitions of absolute iron deficiency (AID) and functional iron deficiency (FID), a total of 252 infusions (33.7%) were administered to patients with AID (defined as ferritin < 30 µg/L), while 496 infusions (66.3%) were given to patients with FID (defined as ferritin ≥ 30 µg/L with Tsat < 20%)

Among subjects with CKD, the leading cause of renal impairment was diabetic nephropathy (30%; *n* = 202). Polycystic kidney disease was present in 6.1% (*n* = 41) of subjects, while glomerulonephritis, hypertensive and ischemic nephropathy, obstruction and reflux nephropathy, unknown CKD aetiology, and CKD of miscellaneous other causes contributed the remaining cases (16%, 15%, 13%, 8%, and 12%, respectively). 

Among the 130 subjects with iron-deficiency anemia (IDA) requiring surgery, gastrointestinal (GI) indications were predominant. Specifically, the distribution was as follows: 24.6% (*n* = 32) had lower GI diseases, 23.1% (*n* = 30) had upper GI conditions, 19.2% (*n* = 25) had genitourinary pathology, 17.7% (*n* = 23) had orthopedic conditions, 9.2% (*n* = 12) had gynecological indications, and 6.2% (*n* = 8) had other reasons for undergoing surgery.

When stratified using the CKD definitions of FID and AID, those with AID (ferritin ≤ 100 μg/L with Tsat < 20%) received 444 (60%) infusions whereas those with FID (ferritin > 100μg/L with Tsat < 20%) received 290 (40%) infusions. It is important to note that patients with ferritin levels exceeding 100μg/L together with Tsat levels above 20% were excluded from this specific analysis, as detailed in Supplementary Table S2.

### 3.1. Haemoglobin Response to Iron Infusions

**Figure 1:**
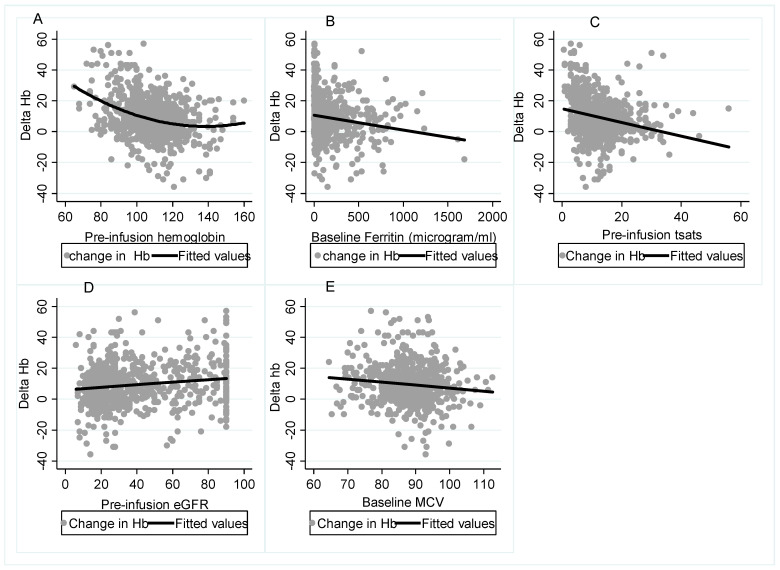
Relationship between ∆Hb and baseline Hb and other iron parameters. In the unadjusted analysis, higher pre-infusion Hb (**A**), ferritin (**B**), Tsat (**C**) and MCV (**E**) were associated with a reduced ∆Hb response, whereas a higher pre-infusion eGFR (**D**) correlated with a better ∆Hb; ∆Hb, change in Hb; Tsats, transferrin saturation; eGFR, estimated glomerular filtration rate.

As shown in Figure 1, when the primary outcome (∆Hb) was considered in relation to baseline parameters, without statistical control of confounders, there appeared to be a clear relationship between better ∆Hb response and greater baseline eGFR, which underpinned the rationale for this study. A reduced ∆Hb response was associated with higher pre-infusion Hb, MCV, ferritin and Tsat.

### 3.2. Propensity Score Matching Analysis Results

**Figure 2:**
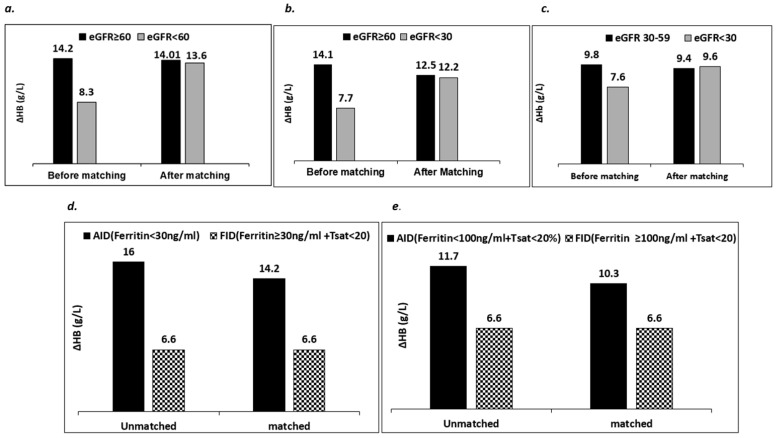
Results of propensity score matched analysis of comparing mean ∆Hb among subjects with different degrees of CKD as well as between subjects with FID and those with AID. (**a**–**c**) display the results of a propensity-score matched comparison among patients with varying degrees of renal impairment, while accounting for the presence of functional iron deficiency (FID) or absolute iron deficiency (AID), as well as factors like age, gender, diabetes, baseline ferritin, baseline Hb, baseline Tsat, and time to repeat Hb. Notably, there was an initially significant difference (∆Hb) before matching, which became non-significant after matching. In (**d**,**e**), the ∆Hb values of those with FID were compared to those with AID, while adjusting for age, gender, diabetes, baseline Hb, and eGFR. The analysis demonstrated a notably superior response in subjects with AID, both before and after propensity score matching.Upon assessing the proportion of individuals who were administered a satisfactory total iron dose (using the Ganzoni formula), it was determined that 86% (716) of the iron infusion met the optimal dosing threshold, while 14% (118) fell below the optimal threshold. Subsequently, we proceeded to analyze the impact of both eGFR categories and the nature of iron deficiency on the change in hemoglobin (∆Hb) exclusively within the subgroup of subjects deemed to have received an optimal iron dose (refer to Figure 3). The outcome closely resembled the main analysis mentioned earlier, revealing that, irrespective of the extent of renal impairment, FID was associated significantly diminished response in ∆Hb, even when optimal iron dose was administered.

The PSM analysis was conducted in two phases (Figure 2). First, we compared responses amongst patients with different stages of renal impairment while adjusting for the presence or absence of FID and other baseline parameters using PSM. In this analysis, 157 subjects with eGFR ≥ 60 mL/min/1.73 m^2^ were matched to 523 subjects with eGFR < 60 mL/min/1.73 m^2^. The groups were matched by age, gender, diabetes, baseline ferritin, baseline Hb, baseline Tsat, FID and time to repeat Hb. 

Before PSM, the mean ∆Hb was significantly higher in those with eGFR ≥ 60 mL/min/1.73 m^2^ compared to those with eGFR < 60 mL/min/1.73 m^2^ (12.1 g/L vs. 8.7 g/L). However, after PSM and adjustment for AID or FID, the difference in ∆Hb between the two groups was no longer apparent (12.4 g/L vs. 12.3 g/L). A similar result was observed when subjects with eGFR ≥ 60 mL/min (*n* = 127) were compared to those with eGFR < 30 mL/min (*n* = 362) (∆Hb, before PSM = 12.3 g/L vs. 7.9 g/L; after PSM = 10.9 g/L vs. 11.2 g/L). The result was no different in subjects with eGFR 30–59 mL/min (*n* = 156) vs. eGFR < 30 mL/min (*n* = 362) (∆Hb before PSM = 10.2 g/L vs. 7.9 g/L after PSM = 9.9 g/L vs. 10.0 g/L). 

The second PSM analysis was focused on evaluating the change in hemoglobin (∆Hb) between individuals with FID and those with AID. The analysis was conducted after ensuring that the two groups were closely matched in terms of age, gender, diabetes, baseline Hb and eGFR. 

PSM analyses were conducted with two different definitions of FID, the traditional definition and the CKD-related definition. With the traditional definition (Ferritin ≥ 30 µg/L and Tsat < 20%), the average rise in Hb before PSM was more than 8 g/L higher in those with AID (*n* = 218) compared to subjects with FID (*n* = 465) (15.0 g/L vs. 6.8 g/L). This difference persisted after matching, which notably included matching for eGFR (14.6 g/L vs. 7.4 g/L). A similar pattern was noted with the CKD-aligned definition of FID (ferritin > 100 µg/L and Tsat < 20%) (∆Hb before PSM = 11.3 g/L vs. 6.7 g/L; after PSM = 10.4 g/L vs. 6.7 g/L).

### 3.3. Multivariate Analysis

Figure 4 and Supplementary Table S3 present the outcomes of the multivariate analysis. Notably, BMI was associated with a positive ∆Hb, [coefficient of ∆Hb (∆Hbβ) = 1.17 (0.37–1.97), *p* = 0.004, per 5 kg/m^2^ increase]. Compared to white ethnicity, black ethnicity was associated with a lower response, ∆Hbβ = −8.12 (−15.02–−1.22), *p* = 0.021. Other predictors of poor response included diabetes, ∆Hbβ = −2.66 (−4.81–−0.51), *p* = 0.015; total iron infusion dose per kg in the 3rd tertile (>20 mg/kg) ∆Hbβ = −3.04 (−5.49–−0.59), *p* = 0.015; higher baseline Hb, ∆Hbβ = −3.22 (−3.94–−2.5), *p* < 0.001; higher baseline MCV, ∆Hbβ = −0.21 (−0.34–−0.08), *p* = 0.002; and the presence of FID ∆Hbβ = −7.61 (−9.94–−5.29), *p* < 0.001 (compared to AID). It is worth noting that FID had the greatest negative impact on response to iron infusion amongst all the predictors that were tested.

### 3.4. Incidence of Adverse Reaction to Parenteral Iron

Infusion-associated reactions occurred in 7 of the 834 infusion episodes (0.8%) (Table 3). Of these, one (0.1%) was a serious allergy (hemodynamic instability requiring hospitalisation but recovered). One was mild localised pruritus, while the other five were ‘Fishbane’ reactions [21,22] manifesting as flushing and mild chest or shoulder pain and which promptly resolved on cessation of infusion. One patient known to have angina had an episode of chest pain. This was judged unrelated to the infusion.

## 4. Discussion

Although anaemia in CKD patients has been attributed to the effects of erythropoietin deficiency and uremia, both of which are dependent on the severity of CKD, the impact of FID alone on Hb response has not been studied. In this study, we used PSM analyses to isolate the effect of FID from that of kidney impairment on Hb response. First, we compared responses among patients with different CKD stages while adjusting for FID. Then we compared the responses between patients with FID vs. AID while adjusting for the degree of renal impairment. Furthermore, combining data from pre-operative and CKD cohorts allowed us to assess Hb responses across a wider range of patients representing all levels of kidney function, including patients without CKD.

Our study revealed a mean ∆Hb of 8 g/L following a mean iron infusion dose of 18.5 mg/kg. This result was significantly higher than that reported by Sivakumar et al. (3.5–4.5 g/L) [23] but lower than the 14–16 g/L reported by the authors of the randomized controlled trial comparing ferumoxytol and ferric carboxymaltose IDA treatment (FIRM) trial [24]. The observed disparities likely stem from differences in the studied populations. Our study cohort encompassed both CKD and non-CKD patients, while Sivakumar et al. focused solely on CKD and peritoneal dialysis patients. In contrast, the FIRM study enrolled individuals with IDA of any aetiology, with a majority experiencing gastrointestinal or abnormal uterine bleeding and possessing largely normal renal function [24].

**Figure 3:**
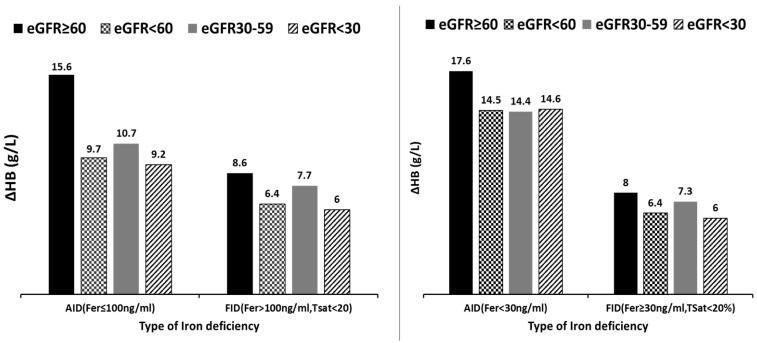
Hb Response stratified by type of iron deficiency and level of kidney function in subjects who received Optimal TDI.

**Figure 4:**
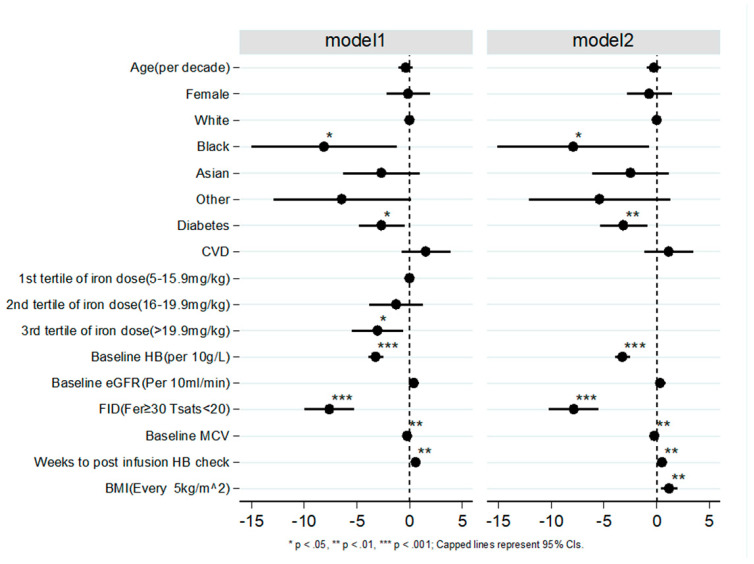
Independent Predictors of Haemoglobin Response (∆Hb) by Multivariate Linear Regression. Model one included the dose/kg tertile and excluded BMI as BMI and dose per kg were correlated. Model 2 contained BMI in place of dose/kg tertile.

In our study, subjects with normal or well-preserved eGFR (no CKD and CKD1–2), showed a better response than those with CKD3–5 (∆Hb = 14.0 g/L vs. 8.3 g/L), which was in keeping with the observations of the pre-surgical anaemia clinic team that stimulated the rationale for this study. However, this difference was no longer apparent after PSM corrected for the effect of FID. Crucially, this pattern held true across the various levels of CKD (Figure 2a–c). On the contrary, when subjects were grouped based on FID vs. AID and matched according to their level of kidney function, there was a significantly better response in those with AID compared to those with FID after matching (Figure 2d,e). Taken together, these findings suggest that response to parenteral iron is far more dependent on the type of IDA (FID vs. AID) than on the severity of kidney impairment. This was also shown in the multivariate analysis in which baseline eGFR did not significantly influence response to iron infusion, whereas the presence of FID was significantly associated with poor response (Figure 4). The strength of these findings was further emphasized after excluding subjects judged to have received a sub-optimal iron dose based on the Ganzoni equation (Figure 3). Our findings confirmed that subjects with severe kidney impairment had Hb responses to IV iron which were the same as their counterparts with mild or no kidney impairment, and this was dependent upon type of iron deficiency, either AID or FID. 

FID, for which a key component of the pathogenesis is due to hepcidin upregulation in chronic inflammatory states (including CKD), is a key factor reducing iron availability for erythropoiesis and inducing erythropoietin resistance [21]. This is probably the reason for the poorer responses in subjects with FID than those with AID irrespective of their kidney function.

The pre-operative intravenous iron used to treat anaemia before major abdominal surgery (PREVENTT) trial found that parenteral iron administered 10–42 days before elective major abdominal surgery was not superior to placebo in preventing death or reducing transfusions in anemic patients [25]. However, the study reported an improved secondary outcome of reduced re-admission in the iron group [25]. The study had limitations, not least that the type of anaemia and the nature of iron deficiency was not defined. Our study suggests that consideration of the nature of a patient’s iron deficiency will identify those who are likely to achieve the desired Hb response after iron infusion. 

Regarding the wider risk predictors of Hb response to parenteral iron, Black ethnicity, lower BMI, diabetes, total iron dose above 20 mg/kg, higher baseline MCV, and presence of FID were independently associated with poor response (Figure 4). 

It was interesting to observe that the dose response to iron infusion appeared to diminish at total doses above 20 mg/kg (Figure 4). The reason for this is not apparent. When we compared the dose received to the dose required according to Ganzoni formula, of the 255 patients who received greater than 20 mg/kg of iron infusion (most in 2 divided doses), only 4 patients received a dose that was equivalent to their iron deficit. These 4 people had a mean Hb rise of 14 g/L (median = 17.5), whereas the remaining 251 patients received a dose greater than their calculated iron deficit (based on Ganzoni formula) and showed a mean Hb rise of 8 mg/kg (median = 7 mg/kg). This implies that patients who receive too much iron for their predicted requirement may have a reduced response. A recent report from Isidori et al. suggested that excess iron with high levels of non-transferrin bound iron (NTBI) could be toxic to the bone marrow through the formation of reactive oxygen species that affect the haematopoesis regulatory proteins [26]. This was not corroborated by a different study that assessed the effect of iron infusion in haemodialysis patients with anaemia and adequate iron stores (the DRIVE study) which showed that increased iron supplementation above target levels improved Hb response [27]. Further studies are needed to ascertain if higher doses of parenteral iron above the calculated deficit adversely affect haematopoetic response.

Our study highlighted the safety of modern parenteral iron preparations. As shown in recent studies, available preparations have been associated with a much lower risk of serious adverse events (SAE) compared to more historic IV iron preparations [28,29]. 

Our findings carry important implications for the management of iron deficient patients. First, patients with pre-operative anaemia and FID are less likely to respond adequately to parenteral iron. These patients should be investigated and treated for reversible causes of FID. Conversely, patients with AID can be anticipated to have a better ∆Hb after parenteral iron, irrespective of their kidney function. Therefore, it is key that the causes of anaemia and the nature of iron deficiency are determined. This should be incorporated into pre- and peri-operative pathways, allowing time for investigation, diagnosis, and treatment. When considering CKD patients, the degree of kidney impairment has less impact on response than the nature of iron deficiency. However, it should be remembered that, although parenteral iron may have a reduced effect on ∆Hb in patients with FID, its effect on other parameters such as cardiac function and exercise tolerance was not addressed in this study and will require further exploration with prospective studies. 

The strength of our study stems from the relatively large number of patients evaluated and the application of PSM to analyse the effect of FID and AID on ∆Hb independent of kidney function and vice-versa. Nevertheless, it is imperative to acknowledge certain limitations inherent to our study. First, the retrospective observational nature of the study inherently exposes the study to potential sampling bias. Although this was mitigated through the meticulous employment of PSM, residual bias cannot be entirely dismissed. Second, a significant limitation arises from the absence of data pertaining to intra-operative blood loss among pre-operative patients who went on to have surgery. This is noteworthy given its potential impact on the post-treatment rise in hemoglobin levels. Thirdly, there is a likelihood that several pre-operative patients with anaemia experienced the resolution of the underlying causes of their anaemia only after surgery; Consequently, a more robust hemoglobin response might have been tempered by ongoing blood loss prior to surgery. Fourth, the variable duration between iron infusion and subsequent hemoglobin reassessment among recipients introduces a potential source of bias. Nonetheless, it is imperative to underline that our analysis addresses this concern through both PSM and multivariate techniques. Further limitations encompass the utilization of the MDRD formula for estimating the glomerular filtration rate (eGFR), which might be less precise than the Chronic Kidney Disease Epidemiology Collaboration (CKD-EPI) formula, particularly at well-preserved kidney function levels. Additionally, the absence of inflammatory markers such as C-reactive protein underscores the real-world nature of our study, which contrasts with prospective designs that could offer a more comprehensive parameter assessment.

## 5. Conclusions

In conclusion, AID and FID are better predictors of Hb response to parenteral iron than kidney function, with the best responses seen in AID patients with lower ferritin and Hb. The presence of FID rather than the level of kidney function per se attenuates the response to parenteral iron. In this study, we found that the intravenous iron used, ferric derisomaltose, was safe and associated with minimal risk of serious adverse reactions, with adequate doses increasing the chance of optimal response. 

## Figures and Tables

**Table 1:** The pre-operative and CKD parenteral iron infusion protocol.

Pre-Operative Regime			CKD Regime				
Pre-Infusion Hb	Parenteral iron Dose by Weight ^α^		Pre-Infusion Hb	Parenteral Iron Dose by Weight ^β^			
	<50 kg	>50 kg		<50 kg	50–69.9 kg	70–100 kg	>100 kg
Hb >80 g/L	500 mg	500 mg	Hb >100 g/L	20 mg/kg	1000 mg	1500 mg	2000 mg
Hb <80 g/L	20 mg/kg	1000 mg	Hb < 100 g/L	20 mg/kg	1500 mg **^µ^**	2000 mg **^µ^**	2000 mg

^α^ Doses up to 500 mg are infused over 15 min while doses exceeding 500 mg are infused over 30 min; ^β^ Doses up to 1000 mg are infused over 15 min while doses exceeding 1000 mg are infused over 30 min; ^µ^ administered in 2 divided doses, maximum individual dose of 20 mg/kg divided into an initial dose of 1000 mg followed by the remaining dose of 500 mg-1000 mg given 1 week later.

**Table 2:** Baseline Characteristics of patient cohort.

	Normal–CKD2 (eGFR ≥ 60) ^¥^	CKD3a (eGFR 45–5 9) ^¥^	CKD3b (eGFR 30–44) ^¥^	CKD4 (eGFR 15–29) ^¥^	CKD5 (eGFR < 15) ^¥^	RRT	TOTAL	*p*-Value
Iron infusions (*n*)	191	64	118	398	57	6	834	-
Number of patients (*n*)	171	49	86	304	38	6	654	-
Age, years (Mean ± SD)	60 ± 18.0	63 ± 17	69 ± 16	70 ± 16	67 ± 13	53 ± 9	66 ± 17	<0.001
Female; *n* (%)	109 (57)	38 (59)	65 (55)	230 (58)	21 (36)	2 (33)	465 (56)	0.058
BMI, kg/m^2^ (Mean ± SD)	28 ± 6.6	27 ± 5.3	29 ± 6.1	30 ± 6.9	28 ± 6.7	29 ± 6.7	29 ± 6.6	0.025
Ethnicity *n* (%)								
- White	152 (80%)	58 (91%)	112 (95%)	347 (87%)	47 (83%)	6 (100%)	722 (87%)	0.024
- Asian	24 (13%)	5 (8%)	5 (4%)	30 (8%)	8 (14%)	0 (0%)	72 (9%)	
- Black	11 (6%)	0 (0%)	0 (0%)	9 (2%)	2 (4%)	0 (0%)	22 (3%)	
- Other	4 (2%)	1 (2%)	1 (0.8%)	12 (3%)	0 (0%)	0 (0%)	18 (2%)	
Diabetes *n* (%)	46 (24)	26 (41)	63 (53)	179 (45)	30 (52)	1 (17)	345 (57)	<0.001
CVD (stroke IHD, HF, PVD) *n* (%)	28 (15)	29 (45)	58 (49)	159 (40)	23 (40)	2 (33)	299 (36)	<0.001
Baseline Hb Median (IQR)	110.0 (99.0–118.0)	110.5 (101.8–124.2)	107.0 (97.2–115.8)	107.0 (99.0–115.0)	105.0 (97.0–114.0)	114.0 (97.5–120.0)	107.0 (99.0–116.0)	0.021
Change in Hb (g/L). Median (IQR)	14 (2–24)	9 (3–15)	7.5 (1–17)	8 (1–14)	4 (−3–10)	6 (1–13)	8 (1–17)	<0.001
Baseline Ferritin (µg/L) Median (IQR)	18.0 (8.0–48.0)	59.0 (26.0–154.0)	63.5 (25.0–171.8)	95.5 (36.0–201.2)	189.0 (102.0–428.0)	195.0 (80.5–312.5)	67.0 (24.0–179.0)	<0.001
Change in ferritin (µg/L) Median (IQR)	204 (109–358)	234 (110–374)	315 (208–500)	341 (191–498)	378 (221–534)	477 (269–829)	359 (170–475)	<0.001
Baseline Tsat (%) Median (IQR)	7 (5–10)	11 (7.5–14.5)	12 (9–14)	14 (11–17)	16 (12–19)	17.5 (15–19)	12 (8–16)	<0.001
Change in Tsat, Median (IQR)	12 (5–18)	13 (8–18)	10 (6–17)	10 (5–16)	8 (5–15)	13 (7–19)	10 (5–17)	0.265
AID	121 (65.1%)	18 (31.6%)	37 (34.9%)	74 (21.3%)	2 (4.3%)	0 (0.0%)	252 (33.7%)	<0.001
FID	65 (34.9%)	39 (68.4%)	69 (65.1%)	274 (78.7%)	44 (95.7%)	5 (100.0%)	496 (66.3%)	<0.001
Baseline MCV, (Normal= 84–105 FL); Median (IQR)	82.2 (76.7–87.6)	89 (84.8–92.7)	89 (84.6–94.4)	90.3 (86.2–93.6)	90.5 (86.8–94.4)	91 (89.7–94.6)	88.7 (83.9–92.8)	<0.001
Baseline MCH, (Normal 27–32 pg); Median (IQR)	26.4 (23.6–28.2)	28.7 (27.0–30.1)	28.8 (27.3–30.7)	29.3 (27.9–30.6)	29.7 (28.4–30.6)	30.6 (28.6–31.7)	28.8 (27.0–30.4)	<0.001

^¥^ indicates units for eGFR in mL/min/1.73 m^2^ calculated using the modification of diet in renal disease equation; values are represented as *n* (%), mean ± SD or median (IQR) unless otherwise stated. SD, standard deviation; IQR, interquartile range; ∆Hb, change in haemoglobin; AID, absolute iron deficiency defined as defined as ferritin < 30; CVD, cardiovascular disease; FID, functional iron deficiency defined as ferritin ≥ 30 and Tsat < 20% BMI, body mass index; Hb, haemoglobin; HF, heart failure; IHD, ischemic heart disease; MCV, mean cell volume (ref. range = 84–105 FL); MCH, mean corpuscular haemoglobin (ref range 27–32 pg); Tsat, transferrin saturation; ¥, indicates units for eGFR in mL/min/1.73 m^2^ calculated using the modification of diet in renal disease equation.

**Table 3:** Safety and tolerability of parenteral ferric derisomaltose.

Infusion Associated Reactions	Severity	Infusion Discontinued
Hypotension, Nausea, and erythema	severe	Yes
Itching	Mild	No
Flushing, back pain	Moderate	Yes
Nausea	Moderate	Yes
Chest tightness	Moderate	Yes
Dizziness and tingling	Moderate	Yes
Shortness of breath, Flushing, and paresthesia	Moderate	yes
Chest pain in a known angina	Moderate	Yes

Serious allergy 0.1% (1/834), labile/Fishbane 0.7% (6/834); mild, local reaction infusion not discontinued; moderate, systemic symptoms but patient not hospitalized; severe, systemic symptoms patient hospitalized.

## Data Availability

All data relevant to the study are included within the article.

## Supplementary Materials

The following supporting information can be downloaded at: https://www.mdpi.com/article/10.3390/biomedicines11092417/s1, Details of propensity score matching, Supplementary Table S1: Propensity score matching analysis of the impact eGFR and type of IDA on ∆Hb following parenteral iron. Supplementary Table S2: Comparison of baseline hemoglobin and iron indices in the subjects stratified by degree of CKD and type of iron deficiency (functional iron deficiency defined as Ferritin > 100 and Tsat < 20%). Supplementary Table S3: Independent predictors of hemoglobin response by multivariate analysis.
