# Supplementary material for: Factors Governing the Erythropoietic Response to Intravenous Iron Infusion in Patients with Chronic Kidney Disease: A Retrospective Cohort Study
Source: Biomedicines. 2023 Aug 29;11(9):2417. doi: 10.3390/biomedicines11092417 (PMC10525177; doi:10.3390/biomedicines11092417)
Supplement: Supplementary file 1 [file biomedicines-11-02417-s001.zip › biomedicines-2543548-supplementary.pdf]

Supplemental Data

Factors governing the erythropoietic response to intravenous iron infusion in patients with chronic kidney disease

Authors

Chukwuma A. Chukwu[1,2] , Helen Gilbody[3], Olivia Wickens[1], Craig Carroll[1], Sunil Bhandari[4], Philip A Kalra[1,2],

1 Department of Nephrology Salford Royal Hospital, Northern Care Alliance NHS Foundation Trust, Salford M6 8HD, UK 2 Faculty of Biology, Medicine and Health, University of Manchester, Manchester M13 9PL, UK 3 College of Medical and Dental Sciences, University of Birmingham, Birmingham B15 2TT, UK; 4 Academic Renal Research Department, Hull University Teaching Hospitals NHS Trust and Hull York Medical School, Kingston upon Hull, Hull HU3 2JZ, UK

Contents

Radius matching for PSM 1

Supplementary Table S1. Propensity score matching analysis of the impact eGFR and type of IDA on ΔHb following parenteral iron. 1

Supplementary Table S2: Comparison of baseline hemoglobin and iron indices in the subjects stratified by degree of CKD and type of iron deficiency with functional iron deficiency defined as Ferritin>100 and TSAT<20% 3

Supplementary Table S3 Predictors of hemoglobin response by multivariate analysis 5

Radius matching for PSM

Patients were matched on the logit of the propensity score using calipers of width equal to 0.25 of the standard deviation of the logit of the propensity score [3,4]. All PSM analyses were conducted with the psmatch2 Stata user-written function for conducting PSM analysis. The mean bias, Rubin's B and Rubin's R were used to assess covariate balance after matching [5]. A Rubin's B<25% and Rubin's R between 0.5-2 are considered to indicate a sufficiently balanced matching [5]. In all the PSM analyses, Rubin's B and Rubin's R suggested high-quality matches as shown in table (Supplementary Table1).

Supplementary Table S1. Propensity score matching analysis of the impact eGFR and type of IDA on ΔHb following parenteral iron.

**Treated vs Control**	**eGFR ≥60[¥] (n=157) vs eGFR<60[¥] (n=523)**	**eGFR ≥60[¥] (n=127) vs eGFR<30[¥] (n=368)**	**eGFR30-59[¥] (n=156) vs eGFR<30[¥] (n=362)**	**AID[α](n=218) vs FID[π] (n=465)**	**AID[β](n=405) vs FID[γ] (n= 272)**
**Matched Covariates**	**Age, sex, diabetes, CVD, baseline Hb, ferritin, TSAT, FID/AID, Duration from**	**Age, sex, diabetes, CVD, baseline Hb, ferritin, TSAT, FID/AID, Duration**	**Age, sex, diabetes, CVD, baseline Hb, ferritin, TSAT, FID/AID,**	**Age, sex, diabetes, CVD, baseline Hb, eGFR, Duration from**	**Age, sex, diabetes, CVD, baseline Hb, eGFR, Duration from**

	infusion to Hb check				from infusion to Hb check				Duration from infusion to Hb check				infusion to Hb check				infusion to Hb check			
**Before & After PSM**	**U**		**M**		**U**		**M**		**U**		**M**		**U**		**M**		**U**		**M**	
**Mean Bias %**	47.9		5.3		50.4		7.1		22.0		1.8		31.8		6.2		27.3		4.4	
**Rubin's B**	146.1		**23.4**		167.8		**24.5**		81.9		**9.7**		87.8		**14.7**		66.8		**10.8**	
**Rubin's R**	1.11		**1.43**		1.21		**1.72**		1.03		**1.34**		0.59		**1.21**		0.61		**1.04**	
**P-value**	0.000		0.892		<0.001		0.968		<0.001		1.00		<0.001		0.270		<0.001		0.894	
**ΔHb (g/L)**	**T**	**C**	**T**	**C**	**T**	**C**	**T**	**C**	**T**	**C**	**T**	**C**	**T**	**C**	**T**	**C**	**T**	**C**	**T**	**C**
**(T:C)**	14.2	8.3	14.0	13.6	14.1	7.7	12.5	12.2	9.8	7.6	9.4	9.6	16.0	6.6	14.2	6.6	11.7	6.6	10.3	6.6

Radius matching within a calliper of 0.25 x SD of the log of propensity scores (0.29). Rubin's B; absolute standardized difference of the means of the linear index of the propensity score in the treated and (matched) control group; Rubin's R, the ratio of treated to (matched) control variances of the propensity score index; A Rubin's B <25% and a Rubin's R between 0.5 and 2 after PSM indicates a sufficiently balanced matching; ΔHB, change in HB; AID, Absolute iron deficiency; α, Absolute iron deficiency defined as ferritin<30µg/l; β, absolute iron deficiency defined as ferritin ≤ 100µg/l & TSAT<20%; C, control; CVD, cardiovascular disease; eGFR, estimated glomerular filtration rate(ml/min/1.73m2); FID, functional iron deficiency; π, functional iron deficiency defined as ferritin≥30µg/l & TSAT<20%; γ, functional iron deficiency defined as ferritin>100µg/l & TSAT<20%; ¥, eGFR in ml/min/1.72m^2, calculated using the modification of diet in renal disease equation: M, matched; T, treated; TSAT, transferrin saturation(%); U, unmatched.

Supplementary Table S2: Comparison of baseline hemoglobin and iron indices in the subjects stratified by degree of CKD and type of iron deficiency with functional iron deficiency defined as Ferritin>100 and TSAT<20%

**Variables**	**TOTAL**		**Normal –CKD2 (eGFR ≥60)**		**CKD3a (eGFR 45-59)**		**CKD3b (eGFR 30-45)**		**CKD4 (eGFR 15-29)**		**CKD5 (eGFR <15)**		**RRT (PD/HD)**	
	**FID**	**AID**	**FID**	**AID**	**FID**	**AID**	**FID**	**AID**	**FID**	**AID**	**FID**	**AID**	**FID**	**AID**
**Age, (yr)**	69±15	65±18	63±16	59±18	58±16	66±17	70±15	69±16	72±13	68±18	67±14	70±13	55±10	44
**BMI - Kg/M^2**	29±7	29±7	26±5	28±7	28±6	27±5	30±8	28±6	30±7	29±7	27±7	28±6	27±7	35
**Baseline Hb(g/L)**	107(98-115)	108(100-117)	111(103-125)	109(98-117)	115(105-128)	109(100-122)	106(97-115)	108(96-116)	107(98-114)	108(100-117)	105(97-114)	105(101-117)	102(91-116)	128
**Baseline Ferritin μg/l**	236(142-399)	28(14-52)	321(144-563)	15(7-28)	180(137-328)	32(16-58)	236(159-349)	28(15-47)	123(60-255)	38(23-61)	307(177-568)	58(36-80)	289(195-443)	58
**Baseline TSAT(%)**	13(10-16)	11(6-14)	9(7-11)	7(4-10)	11(7-14)	11(8-15)	12(10-14)	11(7-14)	14(11-16)	13(9-16)	15(12-17)	13(12-16)	18(16-19)	15
**Baseline MCV**	91(87-94)	86(82-91)	86(81-90)	82(76-87)	90(87-93)	86(82-89)	91(85-96)	87(85-94)	91(86-94)	89(84-91)	91(87-96)	89(86-94)	91(84-95)	101

**Baseline MCH**	30(28-31)	28(26-30)	28(25-29)	26(24-28)	29(28-31)	28(27-30)	30(28-31)	28(27-30)	29(28-31)	29(27-30)	30(28-31)	29(28-31)	31(26-32)	33
**Baseline CRP**	10(4-37)	4(4-14)	32(4-78)	4(4-14)	8(4-27)	4(4-19)	8(4-18)	4(4-11)	9(4-38)	5(4-14)	22(5-34)	10(4-30)	8(4-19)	19
**Iron dose (mg/kg)**	18±4	18±3	17±3	18±2	19±5	19±3	18±4	18±4	18±3	18±4	19±5	17±3	23±4	17
**ΔHb**	7(1-13)	10(2-20)	8(1-16)	16(3-25)	7(1-14)	10(4-16)	5(-2-11)	13(5-18)	7(1-13)	9(1-16)	5(-4-9)	5(-4-8)	13(-1-13)	6
**infusions (subjects)**	290(279)	444(433)	29(28)	151(149)	22(20)	32(35)	41(39)	65(62)	162(157)	181(176)	32(31)	14(13)	4(4)	1(1)

The table shows a significantly lower hemoglobin response in subjects with FID compared to those with AID across all levels of CKD; Values are N, N(%), mean±SD or median(IQR) unless otherwise stated; FID defined as Ferritin>100 and TSAT<20%; BMI, Body mass index; CVD, cardiovascular disease; Hb, hemoglobin; HF, heart failure; IHD, ischemic heart disease; MCV, mean cell volume (Ref-range= 84-105 FL); MCH, mean corpuscular hemoglobin (Ref-range 27-32 pg); TSAT, transferrin saturation; yr(year)

Supplementary Table S3 Predictors of hemoglobin response by multivariate analysis

Variables	Model 1			Model 2		
	**Coefficient**	**96%CI**	**p-value**	**Coefficient**	**95%CI**	**p-value**
Age (per decade)	-0.36	(-1.04-0.33)	0.311	-0.25	(-0.94-0.44)	0.474
Sex	-0.13	(-2.23-1.96)	0.9	-0.7	(-2.82-1.42)	0.515
Ethnicity						
White	0.00	(0.00-0.00)	_	0.00	(0.00-0.00)	
Black	-8.12	(-15.02--1.22)	**0.021**	-7.93	(-15.11--0.75)	**0.031**
Asian	-2.67	(-6.33-0.99)	0.152	-2.45	(-6.08-1.17)	0.184
Other	-6.4	(-12.95-0.15)	0.055	-5.4	(-12.13-1.32)	0.115
Diabetes	-2.66	(-4.81--0.51)	**0.015**	-3.14	(-5.39--0.88)	**0.006**
CVD	1.54	(-0.78-3.86)	0.193	1.15	(-1.18-3.48)	0.333
Iron dose per kg tertile						
5-15.9mg/kg	0.00	(0.00-0.00)	_	_	_	_
16-19.9mg/kg	-1.29	(-3.84-1.27)	0.324	_	_	_
>19.9mg/kg	-3.04	(-5.49--0.59)	**0.015**	_	_	_
BMI (Kg/m^2)	_	_	_	1.17	(0.37-1.97)	**0.004**
Baseline HB	-3.22	(-3.94--2.5)	**<0.001**	-3.25	(-3.98--2.52)	**<0.001**
eGFR (MDRD)	0.4	(-0.06-0.86)	0.091	0.35	(-0.11-0.81)	0.140
Type of Iron deficiency						
AID(Fer<30)	0.00	(0.00-0.00)	_	0.00	(0.00-0.00)	_
FID(Fer≥30 Tsats<20)	-7.61	(-9.94--5.29)	**<0.001**	-7.87	(-10.2--5.54)	**<0.001**
Baseline MCV	-0.21	(-0.34--0.08)	**0.002**	-0.2	(-0.33--0.07)	**0.003**
Weeks to post infusion Hb	0.57	(0.19-0.95)	**0.003**	0.51	(0.13-0.90)	**0.008**

Model one included the dose/kg tertile and excluded BMI as BMI and dose per.kg were correlated. Model 2 contained BMI in place of dose/kg tertile

AID, absolute iron deficiency, CVD, cardiovascular disease; FID, functional iron deficiency; MCV, mean corpuscular volume.
